# A Longitudinal Magnetoencephalographic Study of the Effects of Deep Brain Stimulation on Neuronal Dynamics in Severe Anorexia Nervosa

**DOI:** 10.3389/fnbeh.2022.841843

**Published:** 2022-05-18

**Authors:** Sven Braeutigam, Jessica Clare Scaife, Tipu Aziz, Rebecca J. Park

**Affiliations:** ^1^Oxford Centre for Human Brain Activity, Medical Sciences Division, University of Oxford, Oxford, United Kingdom; ^2^Wellcome Centre For Integrative Neuroimaging, Medical Sciences Division, University of Oxford, Oxford, United Kingdom; ^3^Department of Psychiatry, Medical Sciences Division, University of Oxford, Oxford, United Kingdom; ^4^Oxford Health NHS Foundation Trust, Oxford, United Kingdom; ^5^Nuffield Department of Surgical Sciences, Medical Sciences Division, University of Oxford, Oxford, United Kingdom; ^6^Department of Neurosurgery, Oxford University Hospitals NHS Trust, Oxford, United Kingdom

**Keywords:** anorexia nervosa, deep brain stimulation, magnetoencephalography, treatment, alpha power, N400 & P600, phase-locking

## Abstract

Anorexia Nervosa (AN) is a debilitating psychiatric disorder characterized by the relentless pursuit of thinness, leading to severe emaciation. Magnetoencephalography (MEG)was used to record the neuronal response in seven patients with treatment-resistant AN while completing a disorder-relevant food wanting task. The patients underwent a 15-month protocol, where MEG scans were conducted pre-operatively, post-operatively prior to deep brain stimulation (DBS) switch on, twice during a blind on/off month and at protocol end. Electrodes were implanted bilaterally into the nucleus accumbens with stimulation at the anterior limb of the internal capsule using rechargeable implantable pulse generators. Three patients met criteria as responders at 12 months of stimulation, showing reductions of eating disorder psychopathology of over 35%. An increase in alpha power, as well as evoked power at latencies typically associated with visual processing, working memory, and contextual integration was observed in ON compared to OFF sessions across all seven patients. Moreover, an increase in evoked power at P600-like latencies as well as an increase in γ-band phase-locking over anterior-to-posterior regions were observed for high- compared to low-calorie food image only in ON sessions. These findings indicate that DBS modulates neuronal process in regions far outside the stimulation target site and at latencies possibly reflecting task specific processing, thereby providing further evidence that deep brain stimulation can play a role in the treatment of otherwise intractable psychiatric disorders.

## Introduction

Anorexia nervosa (AN) is a severe eating disorder with the highest morbidity and mortality rate of any psychiatric disorder. In a third of cases, it does not respond to existing treatments and in these individuals, no current psychological or pharmacological treatments are of proven benefit. There is thus a huge unmet need for novel treatments for severe intractable AN, and it is important that these are developed to the highest ethical high standards (Park et al., 2017; Pugh et al., 2018; Pugh, 2019).

Deep brain stimulation (DBS) is a neurosurgical procedure in which electrodes are inserted into specific neural targets with stimulation from an implantable pulse generator, which acts like a pacemaker. It was pioneered by Heath to treat psychiatric disorders, particularly schizophrenia (O’Neal et al., 2017) and was later developed as a treatment for pain (Hosobuchi et al., 1977; Richardson and Akil, 1977). It is now primarily used to treat movement disorders such as Parkinson’s disease (Krack et al., 2019), however, over the last 20 years exploratory studies applying DBS to the treatment of intractable psychiatric disorders have gathered pace. The majority have focused on OCD and depression (Lozano et al., 2008; Cleary et al., 2015; Graat et al., 2017) with some in addiction cohorts (Wang et al., 2018; Vannemreddy and Slavin, 2019) and a very few focusing on AN (Lipsman et al., 2017; Villalba Martínez et al., 2020). The nucleus accumbens (NAcc) within the ventral striatum (VS) has been selected as the target for a number of prior DBS studies in treatment resistant OCD (Denys et al., 2010, 2020; Tyagi et al., 2019) and depression (Malone et al., 2009; Bewernick et al., 2010) because it is a deep brain locus of hedonic pleasure and reward learning (Hill et al., 2014; Berridge and Kringelbach, 2015). There is evidence that disorders of compulsivity, including OCD, addictions and AN, result in part from dysfunctional cortico-striatothalamic reward pathways which contribute to habitual behavior mediated by structures in the striatum (Robbins and Everitt, 1996; Steinglass and Walsh, 2006; Haber and Knutson, 2010; Godier and Park, 2014; Simmler and Ozawa, 2019). In this study, electrodes were implanted bilaterally into the NAcc with distal stimulation at NAcc and proximal stimulation at the anterior limb of the internal capsule (ALIC).

There is incomplete knowledge of the mechanisms underlying DBS, and a paucity of studies in AN. This exploratory longitudinal MEG study thus aimed to enhance the understanding of neuronal markers of DBS action and its possible therapeutic effects. Given the current situation, we feel it is impossible to frame robust *a priori* hypotheses; however, our approach attempts to addresses three broad issues for research deemed to be relevant in this context. Namely, the extent to which DBS: (a) affects brain oscillations far lower than the stimulus frequency; (b) modulates the neuronal response to high vs. low food images; and (c) triggers secondary cognitive processes not directly related to the task demands. To this end, the data analysis and interpretation focused on well established, model-free EEG and/or MEG measure that have proven to be useful for clinically oriented research, such as α-rhythm spectral estimation, event-related amplitudes, and γ-band phase-locking as a measure of neuronal synchrony, network dynamics, and functional connectivity to some extent (Braeutigam et al., 2008; Menassa et al., 2018). Source localization was not considered at this stage, as there is at current very little known about how stimulus artifacts might interfere with the models needed for estimation.

## Material and Methods

### Subjects

Seven patients (six female) with severe, enduring restrictive AN took part in this study. They had all experienced at least three prior inpatient admissions and numerous treatments prior to inclusion in the study. The mean time since disease onset was 21 years (*SD* = 11.8); further demographic and clinical characteristics are detailed in Table 1. The inclusion/exclusion criteria and patient selection process is described in in our published protocol (Park et al., 2018) and accompanying ethical gold standard (Park et al., 2017).

**Table 1:** Demographic and clinical patient characteristics.

**Patient**	**Sex**	**Age**	**Illness duration (years)**	**BMI (historic low)**	**Psychiatric comorbidities**	**Psychotropic medication at time of surgery**	**Inpatient admissions**	**Medical complications**
||1||	F	54	40	13.0	OCD, MDD	Venlafaxine	>3	Osteoporosis
||2||	F	36	13	12.0	OCD	None	>4	Osteoporosis
3	F	28	14	13.0	OCD, MDD, GAD	Sertraline Mirtazepine Pregabalin	>4	Osteoporosis
4	M	38	12	12.0	OCPD Severe recurrent MDD	None	>5	Leukopenia abnormal LFT
5	F	58	36	14.0	MDD	Venlafaxine	>3	Osteoporosis
||6||	F	25	15	13.0	OCD	none	>4	Osteoporosis
7	F	30	17	11.0	MDD	Sertraline	>5	Osteoporosis
								Leukopenia

*BMI, Body mass index; OCD, Obsessive Compulsive Disorder; MDD, Major Depressive Disorder; GAD, Generalized Anxiety Disorder; OCPD, Obsessive Compulsive Personality Disorder; LFT, Liver function tests. Mean age: 38 ± 12.9 years. Mean illness duration: 12 ± 21 years. Mean BMI: 12.6 ± 1. Medication at end of protocol was the same as at time of surgery, but also Patient 5 on Fluoxetine and Patient 2 on Fluvoxamine. Patients who have responded to DBS treatment are indicated by double lines (||)*.

### Protocol Summary

All patients underwent a 15-month protocol, incorporating DBS for 12 months. Patients were assessed monthly using the Eating Disorder Examination (EDE; Fairburn et al., 2008) as the primary outcome measure. Comorbid OCD symptomatology was assessed using the Yale Brown Obsessive Compulsive Scale (YBOCS; Goodman et al., 1989) and mood using the Hamilton Depression Rating Scale HAMD (Hamilton, 1960) and Hamilton Anxiety Rating Scale (Hamilton, 1959). After 9 months of stimulation incorporating dose optimization and stabilization, participants underwent a blinded crossover month: receiving 2 weeks of stimulation or no stimulation consecutively. Treatment response was defined as >35% reduction in EDE at 12 months DBS stimulation. This criteria were chosen to be broadly in line with the definition of response in prior DBS studies using the YBOCS in treatment resistant OCD cohorts (Denys et al., 2010).

### Experimental Design

MEG scans were conducted on five occasions; pre-operatively, post-operatively prior to DBS switch on, in each on/off condition during the blind on/off month, and at the end of protocol. Thus, three OFF MEG scans and two ON MEG scans were acquired in total.

**Figure 1:**
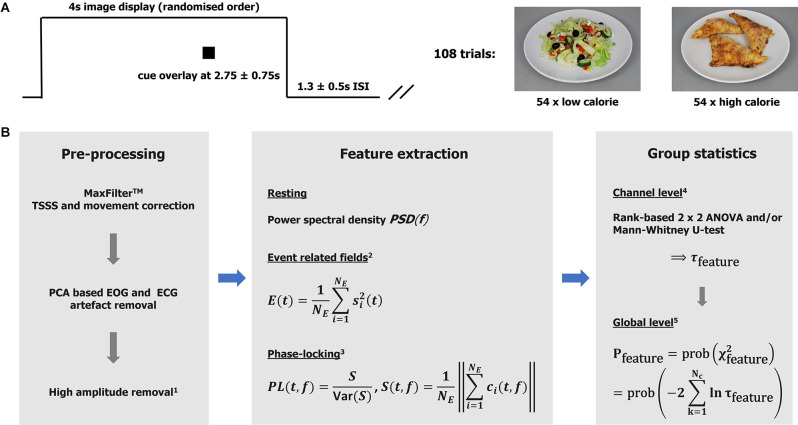
Taskand analytical approach. **(A)** The graph illustrates one experimental trail comprising image display, cue display, andinter-stimulus interval. Sample images of the low- and high-calorie categories are shown on the right. **(B)**Analysis pipeline. MaxFilter^TM^ is a proprietary software of MEGIN (TSSS temporal extension of signal-space-separation). ^1^High amplitudes detection based on global field power. Bad intervals were zeroed (resting data) or removed from the epoch-based analysis (total loss < 5%). ^2^Event related field power was used as a measure of activity. The signals were low-pass (<=30 Hz) filtered, baseline corrected (−100–0 ms), and squared before averaging over trials. *N_e_*: number of epochs. ^3^Phase-locking (PL) based on a Gabor transform with resolutions Δ*t*_80 Hz_ ≈11 ms and Δ*f*_80 Hz_ ≈7 Hz in time and frequency, respectively. The i^th^-epoch Gabor spectral coefficient is denoted by c_i_. The variance of S was estimated using a bootstrap (over epochs) algorithm with 250 repetitions. Note PL is unit-less. The features (measures) are defined for each subject, experimental condition (t, f, or t-f) point, and channel. ^4^The channel statistics yield probabilities τ defined for each (t, f, or t-f) point and channel, given a feature and comparison of interest. ^5^The global (whole head; *N*_c_: number of channels) statistics are defined for each (t, f, or t-f) point. Intervals with *p* <= 0.01 were considered significant and mapped back to the channel level (integrating over time and/or frequency if appropriate) for further consideration.

Whilst in the MEG scanner, participants completed a simple food pictures task. Stimuli were 40 high-resolution (1,034 × 768), standardized digital color photographs of foods divided equally into high-calorie and low-calorie categories (sample images are shown in Figure 1A). The food pictures were presented *via* the Presentation software package and projected onto a screen *via* a projector (viewing distance 1.2 m, screen size 54.5 × 43 cm, resolution 1,280 × 1,024, and frequency 60 Hz). The order of the pictures was randomized and repeated three times. Each picture was on screen for 4,000 ms, with an inter-stimulus interval of 1,300 ± 500 ms, during which a fixation cross was shown centrally.

During each food picture, a small black square appeared centrally between 2,000 and 3,500 ms after stimulus onset. Participants were required to respond by pressing a button as soon as they saw the black square, and these reaction times were recorded *via* a fORP 932 interface box system. The task took approximately 10 min to complete. Participants were asked to think about “how much they wanted to eat each of the foods right now”. This was based on prior research from our group which showed an increased implicit “wanting” (incentive salience) for low-calorie foods compared to high-calorie foods in individuals with AN, with the inverse pattern to that seen in controls (Cowdrey et al., 2013; Scaife et al., 2016). The food images were provided by L. Charbonnier of the Image Sciences Institute, UMC Utrecht, and created as part of the Full4Health project^1^, funded by the European Union Seventh Framework Program (FP7/2007–2013) under Grant agreement no. 266408.

### Data Acquisition

MEG data was acquired using an MEGIN Triux^TM^ Neo system at OHBA. The system provides a total of 306 channels; however, only data from the 204 gradiometers were considered here. The gradiometers are most sensitive to nearby (cortical) sources. The data were sampled at 1,000 Hz (0.03–330 Hz high-pass/anti-alias filter). The electrooculogram (EOG) and electrocardiogram (ECG) were recording using MEG compatible electrodes. Standard MEGIN HPI (Head Position Indicator) was used to track head movements during scan. Binocular eye-tracking data was recorded by means of an EyeLink 1000 (SR Research) device, which was set up and calibrated once the participants were in the scanner.

Note the MEG scanner at OHBA was upgraded from MEGIN (formerly Elekta-Neuromag) VectorView^TM^ to a Triux Neo^TM^ System about halfway through the project. The two helmet systems have the same form factor with identical channel number and type, allowing combined use of data once differences in noise levels have been considered.

### Data Analysis—MEG

The analytical approaches have been discussed in detail elsewhere (Godier et al., 2016; Menassa et al., 2018), and are summarized in form of an annotated analysis pipeline in Figure 1B. For clarification, clusters of significance (“heat” maps) were defined as sets of spatially neighboring channels with a time or time-frequency value better that the statistical threshold (*p* < = 0.01). Note that functional connectivity is defined here as the co-occurrence of (trial-by-trial) stimulus-locked gamma-band responses observed over segregated brain regions. The evaluation of effects at the group and individual levels was based on each cluster’s maximal (significance) channel. It is appreciated that DBS can lead to artifacts that are difficult to correct, and this analysis relied on the effectiveness of the MaxFilter^TM^ algorithm in cleaning up the data (Airaksinen et al., 2012; Litvak et al., 2021). Note all DBS wires were as magnetically silent as possible. Further details are provided in Supplementary Material S1–S3, S7.

Response times (cue) were extracted from the MEG trigger channel and analyzed using same 2 (low calorie, high calorie) × 2 (DBS-OFF, BDS-ON) ANOVA as for the MEG data but without rank transformation. The same statistics was used for the eye data (x and y deflection, pupil size) employing a measure of the variation (span) in the raw data, as reported previously (Godier et al., 2016).

## Results

All experimental sessions were completed successfully between October 2016 and March 2021. The second session for one subject (patient 7), was canceled due to the MEG scanner being replaced during this time.

### Behavioral Response to Deep Brain Stimulation

Three patients met criteria as responders at 12 months (their EDE reduced by over 35%); total EDE scores on these patients reduced by 70%, 44%, and 35.5% respectively over the 12 months of DBS stimulation. In two of these responders, comorbid OCD symptoms, anhedonia and depression also showed significant reduction and all symptoms recurred temporarily during the blind off window. According to this strict definition of response, the remaining four study participants were non-responders to DBS stimulation in terms of their eating disorder psychopathology. However, they all reported some benefits of participation, and all elected to maintain the stimulators at the end of the protocol. Detailed clinical and neuropsychological outcomes are reported in a separate publication (Scaife et al., 2022).

### Behavioral (Experimental) Data

The subjects responded to the visual cue in 92.3 ± 1.2% of the trials in the food task. The target detection failure trials did not show systematic patterns across subjects and were excluded from further analysis. Overall, the mean reaction (response) time across all subjects and sessions was 545 ± 16 ms, where the response to low calorie food images was on average 72 ms faster compared to high calorie foods (*T* = 2.657; *p* < 0.01). Neither calorific content nor DBS condition (on/off) influenced eye movements (and variations in pupil size) during image presentation according to the span measure as detailed above.

### Electrophysiological Data

**Figure 2:**
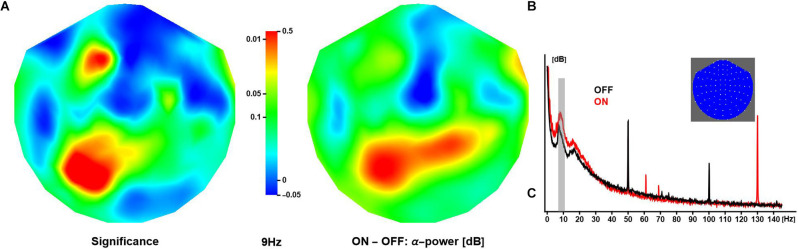
Effect of deep brain stimulation on resting α-activity. **(A)** The panel shows the spatial distribution of significance of the difference in (grand-mean) α-power (at 9 Hz) between conditions, where activity is larger in ON compared to OFF sessions (middle panel). The relative increase in activity over anterior and posterior regions is about 0.3 dB and 0.5 dB, respectively. **(B)** The panel shows OFF and ON spectra obtained in one patient. Note these are raw spectra in which line noise has not been attenuated. The inset shows a 2D projection of the MEG channels (right ear on right, front at top).

In all patients, DBS during the ON sessions was clearly detectable as a spectral peak around 130 Hz, corresponding to the dominant pulse rate of stimulation. Varying across subjects, stimulator models and, to some extent, sessions, other spectral peaks were observed at 47, 54, 78 (two patients only), 108, and 154 Hz amongst others (Figure 2B). DBS spectral contamination within the frequency range typically associated with evoked components (<30 Hz) was small. Note that the neuronal response exhibited substantial inter-subject variability (see Supplementary Material S4 for an illustration); however, robust differences emerged from at the group level, and only averaged data are shown in what follows unless specified otherwise.

### Resting α-Power

Neuronal activity in the alpha range (here: 7–13 Hz) was strongest over posterior/occipital cortices. Compared to OFF, the ON condition elicited significantly stronger α-activity over left posterior and left anterior regions (peak at 9 Hz). Note that some regions showed a reduction in α-activity during ON sessions, however, the difference did not reach significance (Figure 2A).

### Task Related Evoked Fields

**Figure 3:**
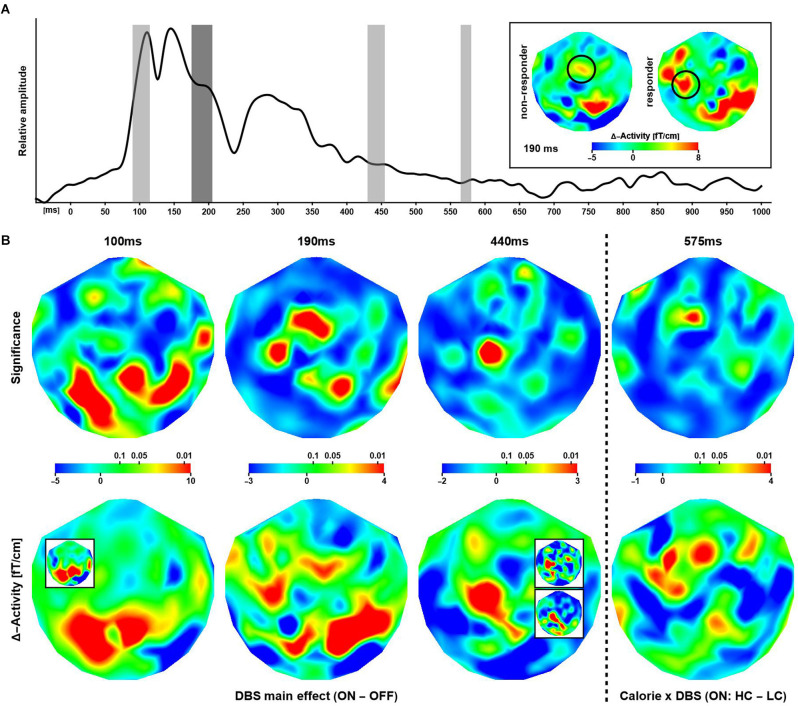
Effectsof deep brain stimulation and calorific value on evoked responses. **(A)** Shown is the global evoked power summed over all subjects, trials, and channels. The gray bars indicate time intervals where a significant modulation of the neuronal response was observed. The inset shows differential (ON–OFF) activity maps corresponding to the effect at 190 ms (highlighted in dark gray in the graph) for non-responders and responders. The circles indicate a putative lateralization of activity in responders during stimulation. **(B)** The maps show the distribution of significance (upper row) corresponding to differential effects of stimulations at three latencies and one interaction effect between stimulation and image type. The (differential) activity maps shown in the lower row are based on evoked power (squared ERF) calculations, however, for visual presentation, the (grand-mean) data have been transformed back to the physical unit (fT/cm) of the MEG gradiometers. Note that some regions of significance exhibit only small differential effects and larger differences in evoked activity are not significant. The insets (bottom row) illustrate the consistency of results when considering subsets of sessions. 100 ms: Session-3 (ON) minus Session-4 (OFF). 440 ms upper: ON minus OFF (sessions 1, 2, 5); lower: ON minus OFF (sessions 3, 4). See text for details.

The global field power (root-mean-square signal) averaged over all patients, channels, and trials (Figure 3A) featured distinguishable, strong peaks for latencies up to about 350 ms after stimulus onset. The overall waveform was consistent with observations made in previous EEG/MEG studies employing similar experimental designs. At longer latencies (>= 500 ms), evoked power was small in general.

**Figure 4:**
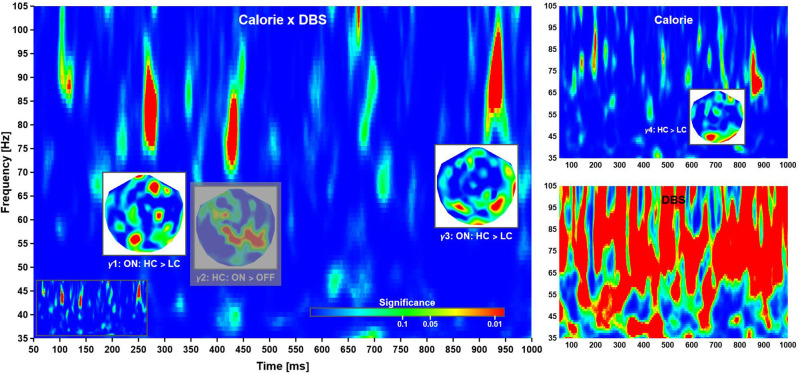
Time-frequency planes. Phase-locking in the high γ-band was modulated by a Calorie × DBS interaction (left), as well DBS (bottom right) and Calorie effects (top right). The insets show the distributions of significance over head for each cluster, where all significant regions within a cluster corresponds to a difference in phase-locking as indicated (at grand-mean level; phase-locking values not shown). The phase-locking indicates time-dependent neuronal networks: anterior-to-posterior, mainly parietal, and mainly occipital. The clusters locate at γ1: 260–285 ms, 75–92 Hz; γ2: 420–440 ms, 71–86 Hz; γ3: 918–945 ms, 83–100 Hz; and γ4: 850–895 ms, 64–83 Hz. Note that γ2 (grayed out) was not further considered (see text for explanation). Also note that deep brain stimulation had a strong influence on phase-locking, thereby rendering the corresponding plane uninterpretable. For completeness, the time-frequency plane of Calorie × DBS significance without overlays is shown as an inset (bottom left).

According to this analysis, the calorific content of the food items presented did not modulate evoked responses at short and intermediate latencies. Beginning with about 500 ms after stimulus onset, several significant phase reversals between evoked waveforms were observed over left anterior temporal and bilateral parietal cortices (not shown). These effects were not reflected by differences in evoked power and did not correlate with cue-induced reaction times. In addition, a significant time-frequency cluster at long latency (γ4: 850–895 ms, 64–83 Hz; Figure 4, top right) reflected an increase in phase-locking following high compared to low calorie food images irrespective of deep brain stimulation. Clusters of significance at early latency (<= 200 ms) were noted but not considered as markers of independent neuronal mechanisms of their short duration and co-occurrence with strong evoked responses.

In contrast, DBS altered evoked power, where three effects at three different latencies were strongest and most robust (Figure 3B columns 1, 2, and 3). At 100 ms, the neuronal responses during ON sessions were significantly stronger than OFF response observed over primarily posterior and to some extend over right temporal regions. Note the strongest differences were seen over the same regions that exhibited the strongest modulation of α-activity in the resting data. At 190 ms after stimulus onset, the pattern of significance shifted anteriorly, where ON responses were stronger compared to activity elicited in the OFF condition. At 440 ms, the differential activity became focal with significance detected only over (left) superior and frontal cortices. Again, ON responses were stronger compared to OFF evoked fields. Note that, in these data, a significant increase in evoked power in OFF compared to ON sessions was not observed.

At 575 ms after stimulus, a significant Calorie × DBS interaction effect on the neuronal response was observed over midline frontal cortices (Figure 3B column 4), where high calorie food items evoked stronger responses than low calorie items, but only in ON sessions. Note that power differences were widespread, but only focally significant.

### Task Related Phase-Locking in the γ-Band

Time-frequency analysis revealed three main clusters of significance corresponding to a Calorie × DBS interaction at short, intermediate, and long latency (Figure 4, left). According to a *post-hoc* analysis, the first cluster of significance (γ1: 260–285 ms, 75–92 Hz) reflected an increase in phase-locking following high compared to low calorie food images in ON sessions. The second cluster (γ2: 420–440 ms, 71–86 Hz) reflected in phase-locking in ON compared to OFF sessions for high calorie food images. Finally, the third cluster (γ3: 918–945 ms, 83–100 Hz) reflected (again) an increase in phase-locking following high compared to low calorie food images in ON sessions.

A (main) effect of deep brain stimulation was observed at most latencies in the γ-band, but no consistent patterns of changes in phase-locking could be identified (Figure 4, bottom right). This was most likely due to some form of “spill-over” from the main pulse frequency as described above. Note this contamination of the phase-locking due to DBS was much less pronounced at lower frequency. Following common practice, we have given significant interaction effects priority over main effects; however, the strong main effect warranted further investigation. To this end, we compared the (local) distribution of probability for the interaction effects with the corresponding time-frequency windows in of the (DBS) main effect. There was some evidence phase-locking cluster γ2 overlapped with the distribution of probability found in the main effect, making it difficult if not impossible to interpret this pattern of activity. For this reason, effect γ2 was excluded from further consideration here, albeit shown in the figure for completeness.

### Effect of Time/Task Repetition

The above analyses were repeated using only data from sessions 3 (“early” DBS on) and 4 (“late” DBS OFF) in patients 1, 3, 4, 6, and 7. Due to the randomization process, patients 2 and 5 had their DBS turned on during sessions 4 and 5 and were excluded here. The data were insufficient for robust estimation of significance; however, the individual and grand mean patterns of neuronal activity and phase-locking agreed with the full data set. An illustration is provided in Figure 3 (panel B bottom row, inset panel 100 ms). Clearly, the pattern of activity observed over occipital cortices at 100 ms after stimulus onset is independent of the temporal order of ON and OFF sessions. In addition, other meaningful groupings of sessions were considered to confirm the observations based on the full data set. For example, a middle (sessions 3 and 4) vs. boundary (1, 2, 5) comparison yielded consistent patterns for the difference between the neuronal activity in the ON and OFF conditions (see Figure 3B for an illustration of the effect at 440 ms).

### Consistency and/or Effects at the Individual Level

The analyses above were complemented by an inspection of individual data sets forming averages over sessions as appropriate, but without using further statistical evaluations. In general, the effects observed at the group level are identifiable at the individual level in most subjects. Specifically, six out of seven patients responded faster to low compared to high calorie foods. It was noted that this effect was strongest in the four patients who had OCD comorbid to AN, but the difference did not reach significance. In case of the electrophysiological data, individual consistency was observed in most patients for each effect, where, in general, the observations based on evoked fields were the most robust (Table 2). There was no indication of a correlation between the neuronal response and AN-OCD co-morbidity.

**Table 2:** Electrophysiological effects at the individual level.

	**Full**	**>=50%**	**<50%**
Resting-α	4	2	1
Evoked-100 ms	5	2	0
Evoked-190 ms	5	1	1
Evoked-440 ms	5	1	1
Evoked-575 ms	5	1	1
γ1	4	2	1
(γ2	4	2	1)
γ3	5	1	1
γ4	4	2	1

*The table indicates number of individual patients who exhibited a given effect observed at the group level. Full: for all significant regions, the corresponding effects were observable at the individual level. >=50%: effects corresponding to at least half of the significant observable at the individual level. In case of an odd number of regions, 50% was obtained by rounding up the numerical half. <50%: less than half (including none) of the regions. For example, there are two regions (anterior and occipital) in the case of resting α-power. Note that γ2 (in parentheses) was not further considered (see text for explanation)*.

### Responders vs. Non-responders

An ancillary analysis was performed revisiting the differential effects above, thereby dividing the data according to (treatment) non-responding and responding subjects. None of the tests reached significance (*p* <= 0.01), however, there was a trend (*p* <= 0.05) for the evoked effect at 190 ms, where the distribution of probability matched the pattern obtained in the main analysis. Accordingly, DBS yielded increased activity over left temporal cortices in responders, whereas such increase, albeit weaker, was observed over more central regions in non-responders (Figure 3A inset; see also Supplementary Material S5, S6).

## Discussion

This longitudinal study recorded the neuronal responses in seven patients with severe enduring AN who underwent implantation of two DBS electrodes targeted at the nucleus accumbens (NAcc) with stimulation at the anterior limb of the internal capsule (ALIC). The clinical outcome in responders, of marked improvement of eating disorder psychopathology (which in two responders was demonstrably due to stimulation given that there was temporary relapse in a blind off period) is a great advance given no other treatments had given them symptomatic relief. Prior studies of non-invasive brain stimulation for eating disorders have shown improvements in mood rather than specific improvements in eating disorder psychopathology (Silva et al., 2019; Sobstyl et al., 2019; Duriez et al., 2020). While some DBS studies in AN report improved BMI and/or eating disorder psychopathology, none have included a blinded on/off period (Lipsman et al., 2017; Liu et al., 2020; Villalba Martínez et al., 2020). The observation that the three responders had an anorexia-OCD comorbidity, with early OCD onset predating the onset of adolescent anorexia, is also novel. In contrast, all non-responders had a later onset of AN, and only one had comorbid OCD also of later onset. As a further important extension to the existing literature, which has focused on behavioral measures and outcomes, the current study provides some insight into the underlying changes in neuronal dynamics due to brain stimulation.

On a related note, interestingly, the data here suggest that the calorific value of food differentially influences both the behavioral and neuronal response under these experimental conditions. For example, at long latencies high calorie food items evoked stronger responses than low calorie items, but only in stimulation ON sessions. The electrophysiological differences at long latencies suggest that calorific value modulates large-scale networks in a possibly reverberant fashion, as suggested by the phase-locking over predominantly posterior regions occurring long after primary visual activation. Note that cluster γ4 is unlikely to be a consequence of the interaction effect represented by γ4, as both are separated by 4–5 times the temporal resolution of the Gabor transform. The meaning of these effects is elusive. However, the findings are broadly in line with a study of visual evoked potentials (VEPs) in healthy volunteers, which showed neuronal responses in healthy volunteers to images of high-energy and low-energy food over distinct time periods, from 160 ms and extending to higher-order processing stages up to 500 ms (Toepel et al., 2009).

These results, however, may not be applicable to individuals with AN, in whom activity in attentional networks seem independent of caloric value, pointing to a generalized attentional bias for food images (Blechert et al., 2011a). Moreover, the neuronal response to high- vs. low-calorie food pictures does not differ in studies comparing acute AN, recovered patients and healthy controls (Godier et al., 2016; Romero Frausto et al., 2021). While it remains unclear how the present observations relate to previous findings, it is likely that a variety of factors such as age, duration and severity of disorder, experimental design and stimulus material play a role. The paucity of relevant studies, in conjunction with vast differences in approach makes it difficult to pinpoint mechanisms as yet. Nevertheless, it is tempting to speculate that the increased reaction time to high-calorie foods impairs attention switching due to a preoccupation with food items which AN patients consider particularly undesirable and threatening to self-control.

A caveat is in order here: It is commonly agreed that functional connectivity established in signal space can be affected by conductivity effects (spreading waves), which, in turn, can make interpretation of results difficult. This concern is mitigated to some extent by: (a) the local sensitivity the gradiometer coils; and (b) the observation of both DBS-only and DBS-stimulus interaction effects that are unlikely to occur simply because of wave conduction. This assumption of local specificity is in line with a recent finding that trial-by-trial (local field) phase-locking correlate with BOLD responses in human auditory cortex (Oya et al., 2018), i.e., localized changes in neural activation measured with high spatial resolution are tied in some way with the stimulus-locked electrophysiological response.

Regarding the effects of DBS, the fact there are changes in neuronal dynamics during ON sessions in these data is not surprising. For 150 years or more it has been known that electrical (including modern magnetic) stimulation to the brain can temporarily change perception, motor function, cognitive processes, and mood states, and that some of the changes may persist for after stimulation ceases (Bortolomasi et al., 2007). Such changes have been confirmed through neuroimaging and hold to varying degree for all known transcranial and intra-cranial methods. However, despite extensive basic science and human studies the mechanisms by which stimulation affects neuronal activity are only partially understood (Thut and Pascual-Leone, 2010; Liu et al., 2020). In the case of DBS, mechanistic theories center on direct inhibition and excitation of neural activity, changes in synaptic filtering, and higher order alteration of information processing (Lee et al., 2019) leading to the hypothesis that the stimulation modulates disease- and/or symptom-related oscillatory neuronal networks (Litvak et al., 2021). At current levels of understanding, this hypothesis seems most relevant to the study of Parkinson’s disease, and other pathologies affecting motor planning and action.

While there is considerable interest in neuroimaging of neuromodulation approaches (Val-Laillet et al., 2015) there are few studies of AN. To the authors’ knowledge, only a single F-18 PET study has been reported suggesting that (glucose) hypermetabolism in the frontal lobe, hippocampus, and lentiform nucleus decreases after deep brain stimulation of the nucleus accumbens in those with AN (Zhang et al., 2013). Consequently, it is largely unknown how electrical and/or magnetic neuromodulation, including DBS, affects the processing of environmental and experimental stimuli in those with AN. The questions raised by the findings in this study will hopefully stimulate further research in this under-researched and poorly understood eating disorder (Godier and Park, 2014; Park et al., 2014).

We can cautiously speculate about the significance of the DBS-related effects observed here: The increase in alpha activity after stimulation appears conceptually related to observations of an increase in alpha in AN patients after refeeding (Hatch et al., 2011). While we are not suggesting that DBS emulates the changes in brain-body state after food intake, it is conceivable that stimulation facilitates some form of relaxed, settled and less obsessive state of mind characterized by reduced cognitive efforts and perhaps visual attentiveness targeted at food items. This would be broadly in line with a growing body of evidence suggesting that an increase in alpha activity achieved through brain-wave neuro-feedback can have positive psychological effects (e.g., limiting anxiety), at least temporarily under certain circumstances (Hardt, 2012).

Regarding evoked fields, we note that the DBS-related effects were observed at latencies associated with the primary visual response, N1-P2 complex, P3, and N400 components, which have been studied extensively across stimulus modalities (Bradley and Keil, 2012). This association by latency and approximate source location suggests that DBS influences a cascade of neuronal mechanism related to primary and secondary visual processing, stimulus evaluation and categorization, selective attention, visual working memory, and semantic processing. Evoked amplitudes are reduced in a variety of mental disorders compared to neuro-typical development (Godier et al., 2016; Braeutigam et al., 2018; Ahtam et al., 2020; Hiluy et al., 2021; Romero Frausto et al., 2021) and DBS may restore a kind of “normality” at the neuronal level. This notion is in line with the hypothesis that electrical neuromodulation shifts abnormal circuits toward a more normative physiological state (Lee et al., 2019).

While these findings are intriguing, important questions remain regarding the specificity of observations. Four, partly overlapping questions appear most pertinent. (1) Are the results related to actual stimulation as opposed to indirect, possibly secondary and/or delayed modulatory mechanism taking place over time between experimental sessions? (2) Are the effects indicative of changes in reward circuitry targeted by DBS? (3) Are the findings task specific? and (4) Are the findings specific to anorexia and possible clinical outcome?

Regarding the first question, the findings here suggest that the changes in neuronal dynamics are directly caused by the presence of stimulation, as evidenced by the results obtained from a “late OFF” vs. “early ON” data split. As a corollary, effects of repetition and habituation seem not to have played a role in these data, which is an interesting observation in its own right, as the test-retest reliability of neuroimaging is still a matter of debate (Garcés et al., 2016; Villalba Martínez et al., 2020). Clearly, care must be taken in making such statements given the small sample size, comprising only a few snapshots of neuronal activity taken within a relatively short period compared to the illness duration in these patients. Nevertheless, a direct link, if confirmed, would be a valuable step towards a better understanding of the role deep brain stimulation can play in treatment-refractory psychiatric disorders (Nuttin et al., 2014; Park et al., 2017). At current, DBS for disorders other than Parkinson’s and similar diseases remains at an exploratory and experimental stage characterized by more unknown than known variables, where there is even some evidence based on randomized controlled (sham) designs suggesting that behavioral response rates do not differ between active and control groups (Dougherty et al., 2015).

The answer to the second question of whether the effects are indicative of changes in reward circuitry targeted by DBS, is a tenuous yes. Although this experimental design did not probe for reward mechanisms directly, a plethora of studies have shown the involvement of γ-band oscillatory dynamics in reward processing in the human ventral striatum (Kalenscher et al., 2010; Lega et al., 2011) reward processing and learning (Marco-Pallarés et al., 2015), and emotional memory (Headley and Paré, 2013). While such findings do not prove that reward mechanisms were modulated in these patients, the current findings in AN are broadly similar in latencies, frequencies as well approximate location. Specifically, the anterior gamma (γ1) might indicate that reward mechanisms were modulated in these patients. The oscillatory dynamics observed here, however, could also reflect reward-independent visual processes of perceptual binding and object recognition (Tallon-Baudry and Bertrand, 1999).

Regarding the third question, the Calorie × DBS effects observed in both the evoked and phase-locked gamma responses suggest that DBS affects neuronal processing in a task specific fashion to some extent. Specifically, the increase in evoked power to high-calorie food images in ON sessions at 575 ms points to processes associated with P600-like waveforms. The P600 is a language-relevant evoked component assumed to reflect, amongst others, processes of revision and re-evaluation in the context of grammatically correct but ambiguous sentences. Although the task used here did not feature language aspects, it is conceivable that the subjects engaged in some form of language-related evaluation and/or semantic processing, of high-vs. low-calorie content of food items under stimulation, a process with might be normal in healthy individuals but is not occurring in AN without intervention (Gonda et al., 2020).

This view is supported by the DBS main effect seen in the evoked responses at N400 latencies. N400-like processes have been shown to reflect contextual integration in a wide range of tasks (Fogelson et al., 2004; Kutas and Federmeier, 2011), further suggesting that these patients might engage in semantic, possibly language-related processes beyond explicit task demands. Note this interpretation relies on the concept of reverse inference, where one postulates the existence of a specific cognitive process based on the observation of certain neuronal markers. At current, reverse inference in the context of language processing in AN is weak; however, existing evidence suggest that the interpretation here is at least a viable speculation. Firstly, it has been shown that cognitive processes associated with the integration of semantic/contextual information can occur regardless of whether they are relevant for task performance (Relander et al., 2009). Secondly, neuronal responses associated with contextual processing have been observed for unusual stimulus pairings, e.g., a piece of classical music serving as a prime for a word (Koelsch et al., 2004). Thirdly, healthy controls showed higher N400 amplitudes semantically incongruent stimuli compared to patient with an eating disorder (Blechert et al., 2011b; strictly speaking, this was only shown for the case of bulimia nervosa). This last point might reinforce the notion that DBS, at least temporarily, makes the neuronal response appear normal (seen here as an increase in N400 in the ON condition).

The answer to the fourth question of specificity to AN, is yet inconclusive. Although a small proportion of the patients studied had shown improvements in general and eating behaviors, the AN-OCD comorbidity existing in the group of responders makes it difficult to establish specificity of outcome. Nevertheless, the results suggest that DBS can yield clinically significant change in eating disorder psychopathology, at least in some patients with treatment intractable anorexia nervosa, over and above possible placebo effects. Specifically, the putative shift in neuronal activity towards more lateral parts of the brain in responders is a promising observation, where a recent MEG study suggests that evoked components between 150 and 250 ms indicate an abnormal motivational response to food in anorexia (Romero Frausto et al., 2021). Still, the precise mechanism of how DBS affects behavior remains elusive, and it is possible that some of the changes in the neuronal response during stimulation could be observed in a neuro-typical sample and, or under different experimental conditions. Moreover, the findings might also reflect persistent cortical dysfunctions observed in patients with AN even after improvements in behavior and weight restoration (Jáuregui-Lobera, 2012).

## Conclusions

This study provides further evidence that DBS can play a role in the treatment of otherwise intractable psychiatric disorders. Despite consistency of electrophysiological effects across all patients, the variation in clinical outcome implies that the underlying mechanism of stimulation are still largely unknown. All the responders developed childhood OCD prior to early adolescent AN onset, whereas non responders had later onset of AN, and only one had later comorbid OCD. It is possible that stimulating the NAcc/ALIC primarily ameliorated OCD symptomatology, and increased flexibility allowing engagement with recovery. Differential outcomes aside, from a system neuroscience perspective, these findings indicate that DBS modulates neuronal process in regions far outside the stimulation target site (Alhourani et al., 2015). Given the extraordinary complexity of the human central nervous system, this is not surprising, but should encourage researchers to study the mechanism underlying direct and reverberant interactions between neuronal systems in more detail.

Despite prior studies suggesting that the NAcc could be a beneficial target for DBS in this patient group, it seems probable that it was not the right one for those who did not respond. This study was limited by a small sample size, a paucity of experimental (task) control conditions, and a lack of source analysis. Nevertheless, our initial findings, especially if confirmed in subsequent studies and paradigms, are a valuable step towards a better understanding of by what means and to what extent DBS can play in treatment-refractory psychiatric disorders (Nuttin et al., 2014; Park et al., 2017).

## Data Availability Statement

The raw data supporting the conclusions of this article will be made available by the authors, without undue reservation.

## Ethics Statement

The studies involving human participants were reviewed and approved by South Central—Oxford A Research Ethics Committee (REC) Ref: 13/SC/0267. The patients/participants provided their written informed consent to participate in this study.

## Author Contributions

RP led on psychiatric aspects of the study, designed the protocol, information sheets, wrote ethics and grant applications. TA led on all surgical aspects of the study. SB and JS acquired the data. SB analyzed the data. SB, JS, and RP drafted the manuscript. All authors contributed to the article and approved the submitted version.

## Author Disclaimer

The views expressed are those of the authors and not necessarily those of the NHS, the NIHR or the Department of Health.

## Conflict of Interest

The authors declare that the research was conducted in the absence of any commercial or financial relationships that could be construed as a potential conflict of interest.

## Publisher’s Note

All claims expressed in this article are solely those of the authors and do not necessarily represent those of their affiliated organizations, or those of the publisher, the editors and the reviewers. Any product that may be evaluated in this article, or claim that may be made by its manufacturer, is not guaranteed or endorsed by the publisher.

## Footnotes

^1^ https://cordis.europa.eu/project/id/266408/reporting
