# Supplementary material for: A Longitudinal Magnetoencephalographic Study of the Effects of Deep Brain Stimulation on Neuronal Dynamics in Severe Anorexia Nervosa
Source: Front Behav Neurosci. 2022 May 18;16:841843. doi: 10.3389/fnbeh.2022.841843 (PMC9178415; doi:10.3389/fnbeh.2022.841843)
Supplement: Supplementary file 1 [file Data_Sheet_1.PDF]

**Supplementary Material**

*A longitudinal magnetoencephalographic study of the effects of deep brain stimulation on neuronal dynamics in severe anorexia nervosa, Braeutigam, Scaife, Aziz, Park.*

S1 A standard peridogram spectral technique was used to estimate α-power. A Hanning window (size = 5000 points) was applied to overlapping data segment before spectral estimation.

S2 A Gabor transform of the following form was used

$$c(t,f) = \sqrt{2\sqrt{\pi}f/K}\int_{-\infty}^{\infty} s(\tau)\, e^{-2\pi i f\tau}\, e^{-2\left(\frac{\pi f(\tau-t)}{K}\right)^2}\, d\tau,$$

where *K* is a free parameter, and *s* is the signal of interest. The transform convolutes a given signal with a time-localised, sinusoidal wave-packet of frequency f with a (half-width) standard deviation equal to $\Delta f = \frac{f}{\sqrt{2}K}$. The temporal half-width is given as $\Delta t = \frac{K}{2\sqrt{2}\pi f}$. Here, K was set to 8.

S3 Statistical analysis pipeline (here: α-power).

1. Preprocessing → 3 x 7 OFF and 2 x 7 ON data sets. Each data sets contains data from 204 (gradiometer) channels.
2. Spectral estimation → (3 x 7 + 2 x 7) x 204 periodograms. Each periodogram has a range 0 – 500Hz (grid size 5000).
3. Channel level statistics (Mann-Whitney U); grouping: 21 vs. 14 data sets → 204 periodogram probability curves (significance as a function of frequency).
4. $\chi^2$-pooled probabilities → global: one probability curve (identification of significant frequencies), or local: 102 curves (distribution of significance over the head).

S4 Individual evoked power time courses averaged over all channels (there are 7 curves in each condition). There is considerable inter-subject variability in these data, although robust effects are observable at the group level. ON/OFF refers to DBS stimulation at time of MEG recording. HC/LC: images of high/low-calorie food stuff.

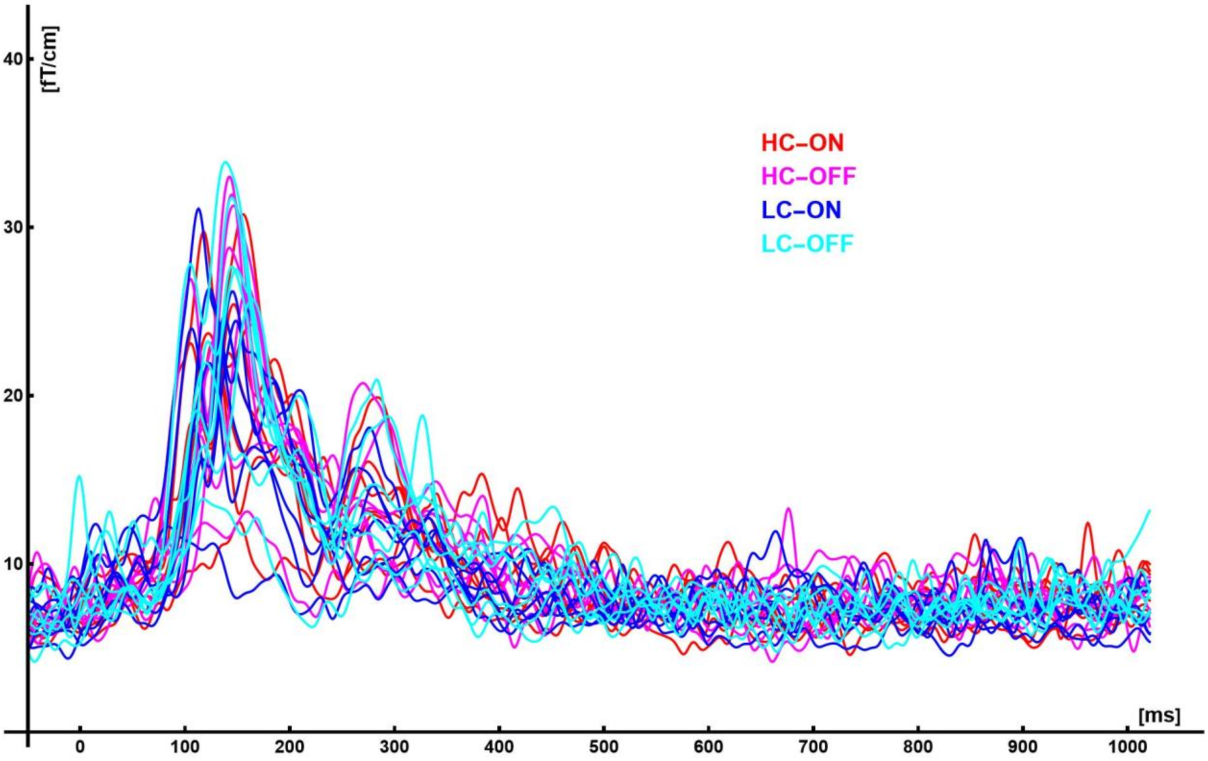

S5 Significant difference between responders and non-responders. Shown are the topographic maps presented in Fig. 3, A (inset) together the corresponding distribution of significance over the head. Note that a) significance is not as strong as for other comparisons in this study, and b) the sign (of effect) can vary from one region of significance to the next. Map based on a Mann-Whitney U-test applied to ON-OFF (difference) power curves. The sensor positions are shown for guidance.

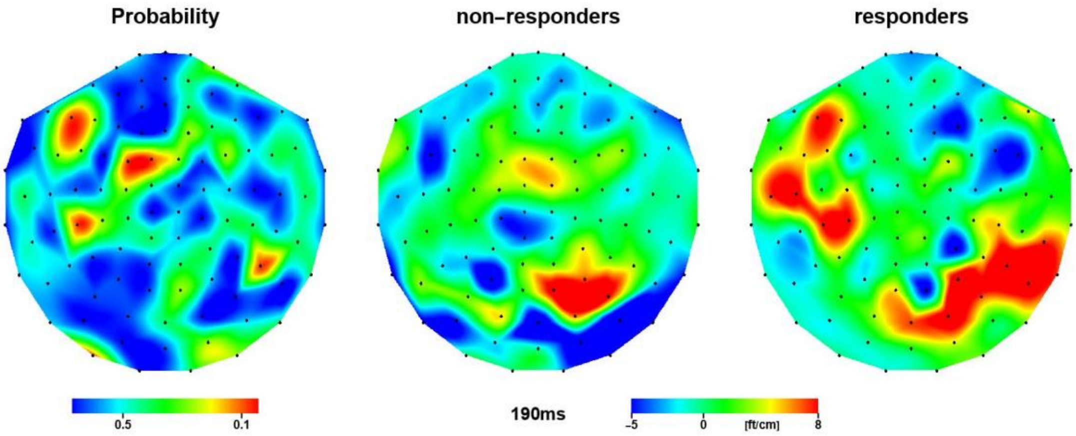


S6 Individual evoked, differential (ON- OFF) power at 190ms after stimulus onset. Inter-subject variability is substantial; however, the individual maps suggest a (left) lateralization of response in responders compared to non-responders.

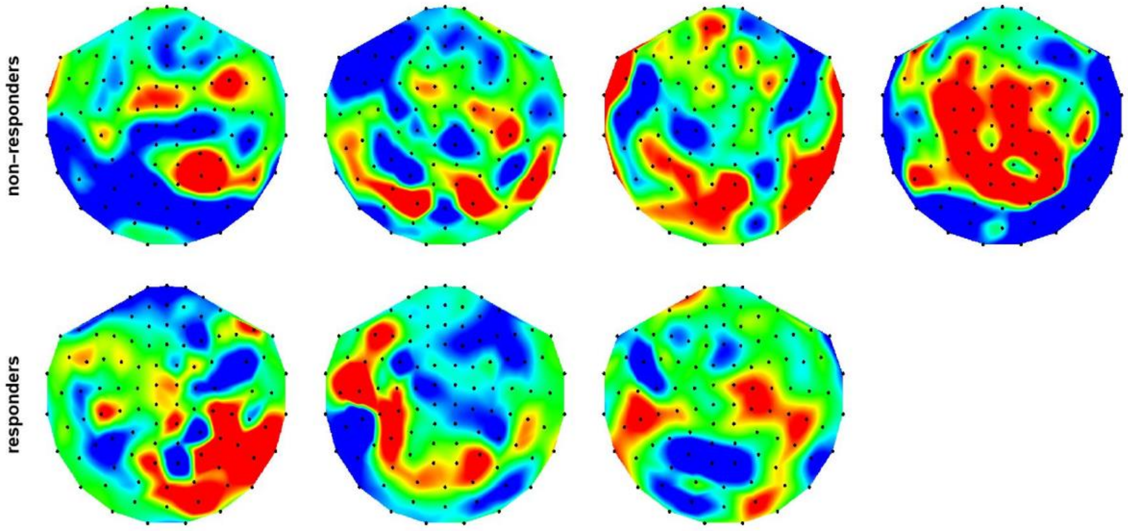

S7 Sensor space analysis. We opted to restrict the analysis to sensor space because a) source estimation under DBS stimulation is still a matter of debate (as mentioned in the text), where different algorithms can yield vastly different results depending on how artefacts are treated, b) phase-locked dynamics (which is a proxy of functional connectivity) is an emergent feature difficult to localise, c) planar gradiometer as used here have high special sensitivity (loosely speaking, act as 0-order beamformers), thereby facilitating approximate localisation of effects over the head, and d) most of the directly relevant literature is based on EEG, where either no or only rudimentary source estimation was performed.
